# Exploring the association between antidepressants, progression and mortality in Huntington’s disease

**DOI:** 10.1093/brain/awag009

**Published:** 2026-01-21

**Authors:** Duncan McLauchlan, Cheney Drew, Peter Holmans, Anne Rosser

**Affiliations:** Centre for Neuropsychiatric Genetics and Genomics, Division of Psychological Medicine and Clinical Neurosciences, School of Medicine, Cardiff University, Cardiff CF24 4HQ, UK; Department of Neurology, University Hospital Wales, Cardiff CF14 4XW, UK; Advanced NeuroTherapies Centre, Hadyn Ellis Building, Cardiff CF24 4HQ, UK; Centre for Trials Research, Cardiff University, Cardiff CF14 4YS, UK; Centre for Neuropsychiatric Genetics and Genomics, Division of Psychological Medicine and Clinical Neurosciences, School of Medicine, Cardiff University, Cardiff CF24 4HQ, UK; UK Dementia Research Institute at Cardiff University, Cardiff CF24 4HQ, UK; Centre for Neuropsychiatric Genetics and Genomics, Division of Psychological Medicine and Clinical Neurosciences, School of Medicine, Cardiff University, Cardiff CF24 4HQ, UK; Advanced NeuroTherapies Centre, Hadyn Ellis Building, Cardiff CF24 4HQ, UK; UK Dementia Research Institute at Cardiff University, Cardiff CF24 4HQ, UK; Cardiff Brain Repair Group, School of Biosciences, Cardiff University, Cardiff CF10 3AX, UK

**Keywords:** propensity score, neurodegeneration, Huntington’s disease, depression, antidepressant

## Abstract

Psychiatric symptoms are very common in Huntington’s disease. In keeping with other neurodegenerative diseases, there are concerns that antidepressants might worsen disease progression. Previous work on antidepressant effects in Huntington’s disease has been limited by confounding by indication, small sample sizes, short follow-up or a combination of these. We leveraged data from the ENROLL-HD (25 550 participants) cohort to determine whether symptoms associated with antidepressant initiation are associated with faster disease progression and whether antidepressants have an impact on disease progression and mortality in people with Huntington’s disease experiencing these symptoms.

Initially, we determined the commonest indications for antidepressant prescription in people with Huntington’s disease. We selected adults with Huntington’s disease (age ≥18 years, with genetically confirmed Huntington’s disease), not on antidepressants and free of antidepressant-indication symptoms at baseline (*n* = 6166) and used linear mixed models to determine the association between symptoms listed as indications for antidepressant prescription and disease progression and mortality. Using propensity score weighting, we selected adults with Huntington’s disease who remained antidepressant naive until an episode of antidepressant-indication symptoms (*n* = 1877) and compared disease progression and mortality between those starting an antidepressant (*n* = 194) before the next follow-up versus those who did not (*n* = 1683). Outcomes were disease progression, measured by the composite disease score in ENROLL-HD, and mortality.

Depression and anxiety accounted for >80% of indications for antidepressant prescription in people with Huntington’s disease: episodes of depression/anxiety (experienced by 3131/6166) were associated with increased composite disease score progression from 0.46 to 0.52/year (*P* = 3.1 × 10^−11^) and increased mortality (hazard ratio = 1.5, *P* = 9.4 × 10^−6^). In people with Huntington’s disease with new depression/anxiety free of antidepressants at symptom onset, antidepressant initiation (*n* = 194/1877) reduced composite disease score decline from 0.89 to 0.53/year (*P* = 0.002) and reduced all-cause mortality (hazard ratio = 0.38, *P* = 0.04). An exploratory analysis of antidepressant classes showed that tricyclic antidepressants reduced suicide and non-suicide mortality; selective serotonin reuptake inhibitors and atypical agents reduced suicide risk, whilst serotonin noradrenaline reuptake inhibitors reduced non-suicide-related mortality.

Depression and anxiety are associated with more rapid disease progression and increased mortality in Huntington’s disease. In people with Huntington’s disease affected by depression and anxiety, antidepressant initiation slows disease progression and reduces mortality risk, with preliminary evidence of antidepressant class-specific reduction in both suicide and non-suicide mortality risk. This finding warrants further investigation in both Huntington’s disease and other neurodegenerative diseases.

## Teaser

By analysing data from the ENROLL-HD study, McLauchlan *et al.* show that in people with Huntington’s disease affected by depression or anxiety, initiation of antidepressants slows disease progression and reduces mortality risk.

## Introduction

Psychiatric symptoms are very common in Huntington’s disease (HD), which is a progressive neurodegenerative disorder focused on cortico-striatal networks that is caused by a CAG repeat expansion in the huntingtin (*HTT*) gene.^1^ The commonest psychiatric symptoms in HD are depression, irritability and apathy.^2^ These symptoms have a significantly higher impact on function and quality of life in HD than do motor impairments.^3^ Consequently, antidepressants and other psychoactive medications are very frequently prescribed to patients with HD.^4^

The evidence base supporting the use of psychoactive medication in HD is limited; no randomized controlled trials of antidepressants for depression as a primary outcome in HD have been performed or are listed on trial registries. Furthermore, evidence from other neurodegenerative diseases, such as Alzheimer’s disease, suggest that antidepressants are ineffective for depression,^5^ and psychoactive medication is associated with higher all-cause mortality in people with dementia.^6^ In HD specifically, recent work has suggested faster disease progression in patients with HD treated with antidepressants.^7^

However, observational studies showing excess mortality and/or faster disease progression in neurodegenerative diseases have not addressed confounding by indication. Prescription of antidepressants and other psychoactive medications might reflect more severe clinical symptoms (and potentially more severe neurodegeneration) necessitating more aggressive symptomatic treatment. Further to this, previous studies showing worsening clinical measures of disease progression in HD have not determined the possible effect on mortality.

This work addresses the potential effects of antidepressants on disease progression by using data drawn from the largest observational study of HD: ENROLL-HD.^8^ ENROLL-HD is a large international observational study of HD, in which participants at all disease stages are followed longitudinally using a range of clinical assessments and self-report scales.

In this work, first we aimed to determine the frequency of common symptomatic indications for antidepressant initiation. Second, we tested for an association between new episodes of these symptoms with both disease progression and mortality to test for confounding by indication. Finally, in antidepressant-naive participants with new episodes of symptoms associated with antidepressant initiation, we compared both disease progression and mortality between those starting an antidepressant versus those who did not.

## Materials and methods

For this study, we used data from the ENROLL-HD observational study.^8^ ENROLL-HD is an ongoing, worldwide observational study of people with HD and familial controls. Information is collected yearly, with formal assessments of cognitive, psychiatric and motor symptoms, in addition to information on co-morbidities, medication, substance misuse and mental health (suicide attempts, admission to an inpatient mental health facility). The most recent release [periodic dataset 6 (PDS6)] included data from 25 550 individuals, with the earliest data being collected in 2012 (mean visit 3.06, visit range 1–15; including unscheduled visits). Death and suicide are captured as significant events in ENROLL-HD. ENROLL-HD adhered to the Declaration of Helsinki, and all recruited patients were formally consented. Both studies received institutional approval from UK research ethics committees.

We included all patients aged ≥18 years, with a confirmed genetic diagnosis of HD. We excluded any HD participants already receiving antidepressants at the baseline study visit to compare the trajectories of disease progression after starting an antidepressant versus not starting one. We determined antidepressant use in the dataset as WHO ATC code N06A.

Psychiatric symptoms are measured by the Problem Behaviours Assessment (short form^8^ ; PBAs) Hospital Anxiety and Depression Scale (HADS^8^) in ENROLL-HD. The PBAs has multiple subscales measuring depressed mood, anxiety and nine other psychiatric symptoms common in HD. Each symptom is scored from zero to four on both severity and frequency, the product of severity and frequency ranges from 0 to 16. The HADS depression score (0–21) and anxiety score (0–21) are self-report scores, with higher scores indicating more severe symptoms. Given that the correlation coefficient between the scales is only moderate in people with HD in ENROLL-HD (0.43, *P* < 2 × 10^−16^), we determined an episode of psychiatric symptoms as the relevant PBAs subscale >4, or relevant HADS subscale >7. Both scales have been validated in people with HD, including participants with cognitive impairment.^9,10^

As clinical outcome variables in ENROLL-HD, we used the most reliable disease progression measure: composite disease score.^11^ This comprises a combination of two cognitive task scores [Stroop Word Reading Task (SWRT) and Symbol Digit Modality Task (SDMT)^8^], the functional scale (total functional capacity) and the motor score from the Unified Huntington's Disease Rating Scale^12^ (UHDRS motor score). The composite disease score becomes more negative with increasing disease progression.

Mortality and suicide are captured in ENROLL-HD as specific events, and the time in days from baseline study visit is recorded.

### Statistical approach

All analyses were conducted using R, an open-source statistical analysis package, using the svy2lme, lme4, survey, Tipr, Sensemakr and Twang packages.

Initially, we determined the commonest indications for antidepressant initiation, listing all indications from the following categories: depression, anxiety, irritability/aggression, psychosis, apathy, other psychiatric symptom, sleep, motor symptom of HD, pain, systemic illness and unclear (Supplementary material, Methods), and selected the most frequent indications for subsequent analyses.

To test for an association between indications for antidepressant initiation and clinical disease progression, we constructed a linear mixed model, with an outcome of the composite disease score, and included baseline composite disease score, baseline psychiatric scores, number of antidepressant prescriptions, comorbidity, addiction, psychoactive drug use, age, sex and CAG repeat length (number of CAG repeats: NCAG) as covariates. The independent variable was defined as episodes of indications for antidepressant initiation (modelled as a time-dependent variable) in ENROLL-HD after baseline; we included subject as a random effect on intercept and slope. As exploratory analyses, we tested for an association between individual symptoms scores modelled as time-dependent variables and clinical disease progression.

We used propensity scoring to determine the effect of antidepressants on disease progression. To avoid confounding by indication (antidepressants are most commonly prescribed to patients with more severe symptoms, who may have worse disease progression), we selected antidepressant-naive patients from ENROLL-HD with incident episodes of indications for antidepressant initiation, throughout the study, and compared subsequent disease progression in those who started an antidepressant before the next follow-up with participants who did not, in an intention-to-treat analysis. In the propensity scoring model, we included: age; sex; total number of antidepressant changes; baseline scores for PBAs depression, PBAs suicidal ideation, PBAs anxiety and PBAs irritability; concurrent other psychoactive medication use (anti-dopaminergic medication, mood stabilizers or benzodiazapines); significant mental health event (suicide attempt or psychiatric hospital admission); multiple comorbidities (more than five); and baseline composite disease score. All variables were included in the outcome model in addition to the inverse propensity score in a doubly robust process, as described by Funk *et al*.^13^ We used linear mixed models to determine the relationship between antidepressant use and change in the composite disease score over time. We fitted Cox proportional hazard models to determine the effect of antidepressants on all-cause mortality. As an exploratory analysis, we fitted Cox proportional hazards for antidepressant effect on suicide and non-suicide death. As an additional sensitivity analysis, we determined the effect of antidepressant exposure (duration of antidepressant treatment) on any significant findings from the intention-to-treat analysis. As an exploratory analysis, we determined the effect of individual antidepressant classes on disease progression and mortality. We used the nnet and simputation packages to compare missing data between treatment groups and impute missing data. Using the tipr and sensemakr packages in R, we conducted a tipping-point analysis as described by D’Agostino McGowan.^14^ To determine the effect of potential unobserved confounding on our findings, we calculated the partial *R*^2^ [partial *R*^2^ = *t*^2^ / (*t*^2^ + degrees of freedom)] of an unobserved confounding variable with both the outcome and exposure that would eliminate the association between antidepressant treatment over time and composite disease score. We then plotted this partial *R*^2^ against exposure and outcome and compared this with the partial *R*^2^ of known covariates to determine the likelihood of such a confounder existing. As an additional sensitivity analysis, we calculated the robustness value^14^ [reported as the percentage of residual variance in outcome explained by an unobserved confounder added to the model; robustness value = ½{*f* + 4*f* − *f*}, where *f* equals the *t*-statistic of the exposure (antidepressant treatment over time) divided by degrees of freedom]. The robustness value was compared with the percentages of outcome variance explained by the other covariates in the model. Missing data did not differ between treatment groups (Supplementary material, Methods). We used a hierarchical approach to account for multiple comparisons: (i) testing for an association between psychiatric symptoms with disease progression and mortality to determine whether confounding by indication was present; (ii) testing the association of antidepressants on disease progression; and (iii) testing the association between antidepressants and mortality.

## Results

### Antidepressant indication

The commonest indications for antidepressant use were anxiety and depression, accounting for >80% of listed indications (Table 1). We therefore selected incident episodes of depression or anxiety (psychiatric symptoms) after study entry as the indication for antidepressant initiation.

**Table 1:** Indications for antidepressants

Indication	*n*	Percentage
Depression	16 405	67.74
Anxiety	3134	12.94
Sleep	2052	8.47
Irritability	990	4.09
Other psychiatric	633	2.61
Pain	323	1.33
Apathy	193	0.80
Systemic illness	184	0.76
Unclear	176	0.73
Motor symptoms of HD	76	0.31
Psychosis	50	0.21

Some prescriptions had multiple indications. HD = Huntington’s disease.

### Association between psychiatric symptoms and disease progression

**Figure 1:**
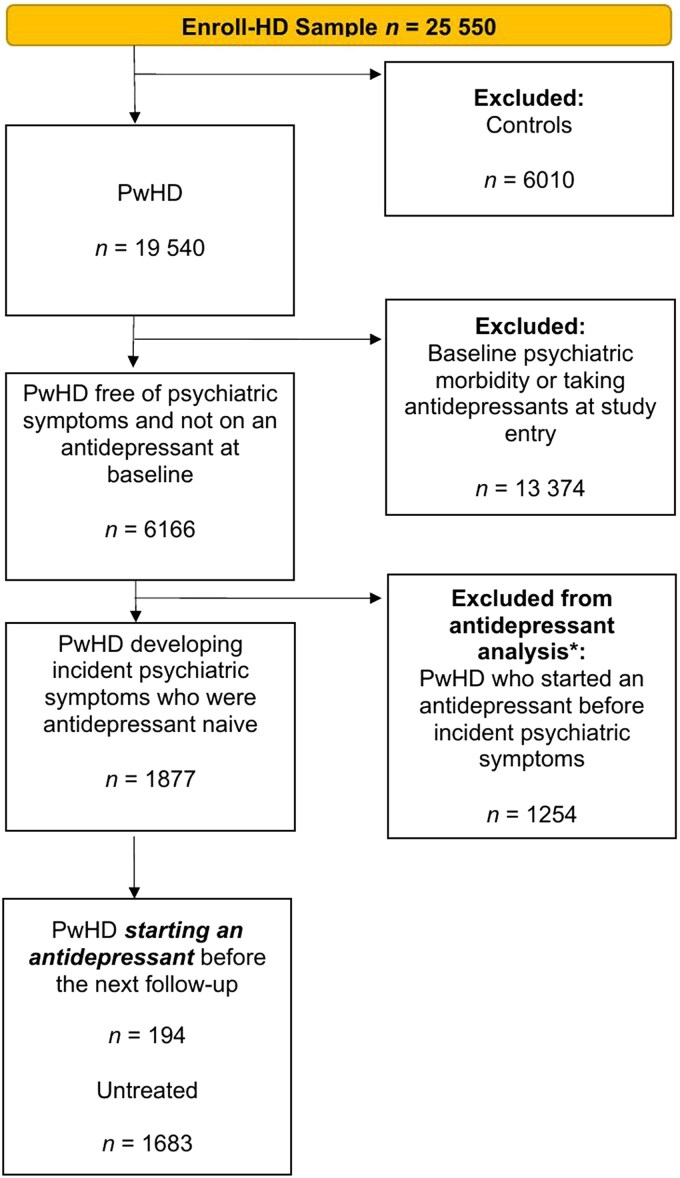
**PRISMA diagram for sample construction from Enroll-HD.** Adapted from Page *et al*.^15^ *These study participants were included in the analyses to determine the effect of psychiatric symptoms on disease progression and mortality. PwHD = people with the repeat expansion for Huntington’s disease.

In ENROLL-HD, 3131/6166 antidepressant-naive participants with HD who were free of psychiatric symptoms at baseline experienced an episode of depression or anxiety during the study (a PRISMA-style^15^ diagram explaining inclusion is shown in Fig. 1). The group with psychiatric symptoms were older, more likely to be female, with more advanced disease and larger NCAG (Table 2).

**Table 2:** Demographics and psychiatric symptoms in the ENROLL-HD cohort

Parameter	Psychiatric symptoms present *n* = 3131/6166	Psychiatric symptoms absent *n* = 3035/6166	*P*-value
Age, years	47.93 (13.81)	47.37 (14.5)	0.034
Sex, female, %	55.55	50.25	*<*0.001
Baseline composite disease score	12.2 (5.25)	13.15 (4.84)	*<*0.001
Baseline PBA depression score	1.21 (1.6)	0.3 (0.72)	*<*0.001
Baseline PBA irritability score	1.43 (2.26)	0.82 (1.8)	0.01
Baseline PBA anxiety score	1.52 (1.68)	0.4 (0.82)	0.01
Baseline PBA suicidality score	0.06 (0.4)	0.01 (0.15)	*<*0.001
Number of antidepressants	0.69 (1.05)	0.45 (0.88)	*<*0.001
Previous mental health event	0.24 (0.43)	0.13 (0.34)	*<*0.001
History of addiction	0.49 (0.5)	0.46 (0.5)	*<*0.001
Psychoactive drug	0.4 (0.49)	0.28 (0.45)	*<*0.001
NCAG	43.29 (3.45)	43.14 (3.64)	0.1
Comorbidities	0.26 (0.44)	0.18 (0.38)	*<*0.001

Data are presented as the mean (standard deviation). NCAG = number of CAG repeats; PBA = Problem Behaviours Assessment.

**Figure 2:**
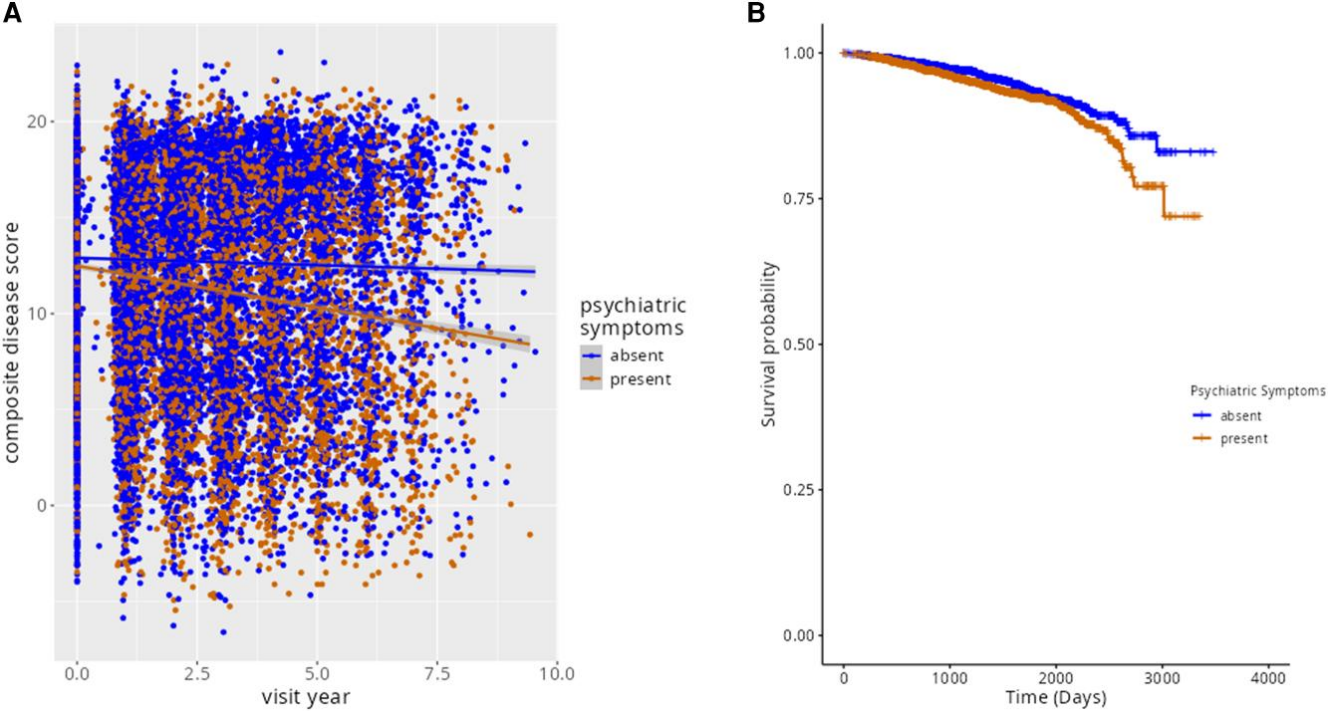
**The association between psychiatric symptoms with disease progression and mortality.** Scatter plot and Kaplan–Meier plot showing the associations between disease progression (**A**) and survival of participants (**B**) experiencing psychiatric symptoms (3131/6166) compared with those not (3035/6166).

The linear mixed model examining the association between psychiatric symptoms (episode of anxiety or depression) and the composite disease score showed that having psychiatric symptoms was associated with faster progression. The composite disease score deteriorated by 0.46/year across all participants. A single episode of psychiatric symptoms was associated with faster deterioration of 0.06/year [95% confidence interval (CI) 0.042, 0.078; *P* = 3.1 × 10^−11^; Fig. 2A and Supplementary Table 1]. As an exploratory analysis, the individual symptom score for depression was also significantly associated with more rapid progression of the composite disease score, but anxiety only approached significance (*P* = 0.056; Supplementary Tables 2 and 3).

All-cause mortality risk was associated with the presence of psychiatric symptoms [hazard ratio (HR) = 1.5, *P* = 9.4 × 10^−6^; Fig. 2B and Supplementary Table 4]. To determine whether the association between increased mortality risk and psychiatric symptoms related to increased suicide rate, as an exploratory analysis mortality was separated into suicide and non-suicide mortality. These models showed that psychiatric symptoms were associated with increased non-suicide mortality but not with suicide risk (Supplementary Tables 5 and 6). Exploratory analyses of individual symptoms scores suggest that depression was more strongly associated with mortality than anxiety (Supplementary Tables 7 and 8).

### Examining the effect of antidepressant use on disease progression

In ENROLL-HD, 1877 antidepressant-naive participants (who had been previously free of depression and anxiety) experienced a new episode of psychiatric symptoms, and 194 were treated with antidepressants (10.33%). The treated group were significantly different from the untreated across a number of variables: the treated group were older, with more advanced disease, higher scores on all psychiatric variables (depression, suicidality, anxiety and irritability), more psychoactive drug use, more frequent antidepressant treatments across the study and more frequent mental health events, but smaller NCAG. After the propensity scoring process, the groups differed only on baseline composite score, but this difference did not meet the threshold for significant imbalance in propensity weights^16,17^ (Kolmogorov–Smirnov statistic < 0.1 and standardized mean difference < 0.2; Table 3 and Supplementary Table 9).

**Table 3:** Demographics and antidepressant treatment in the ENROLL-HD cohort

Parameter	Antidepressant treatment *n* = 194/1877	Control *n* = 1683/1877	Unweighted *P*-value
Age, years	52.13 (11.77)	49.91 (13.62)	*<*0.001
Sex, female	0.57 (0.49)	0.55 (0.50)	0.25
Baseline composite disease score	10.77 (5.11)	11.06 (5.70)	0.16
Baseline PBA depression score	4.43 (3.79)	3.55 (3.50)	*<*0.001
Baseline PBA irritability score	3.15 (3.25)	2.83 (3.25)	0.01
Baseline PBA anxiety score	5.89 (3.97)	5.50 (3.64)	0.01
Baseline PBA suicidality score	0.86 (2.37)	0.34 (1.35)	*<*0.001
Number of antidepressants	2.78 (1.81)	0.79 (1.29)	*<*0.001
Previous mental health event	0.52 (0.50)	0.32 (0.47)	*<*0.001
History of addiction	0.57 (0.49)	0.55 (0.50)	0.16
Psychoactive drug	0.76 (0.43)	0.53 (0.50)	*<*0.001
NCAG	43.08 (2.60)	43.37 (3.35)	0.01
Comorbidities	0.41 (0.49)	0.32 (0.47)	*<*0.001

Data are presented as the mean (standard deviation). NCAG = number of CAG repeats; PBA = Problem Behaviours Assessment.

**Figure 3:**
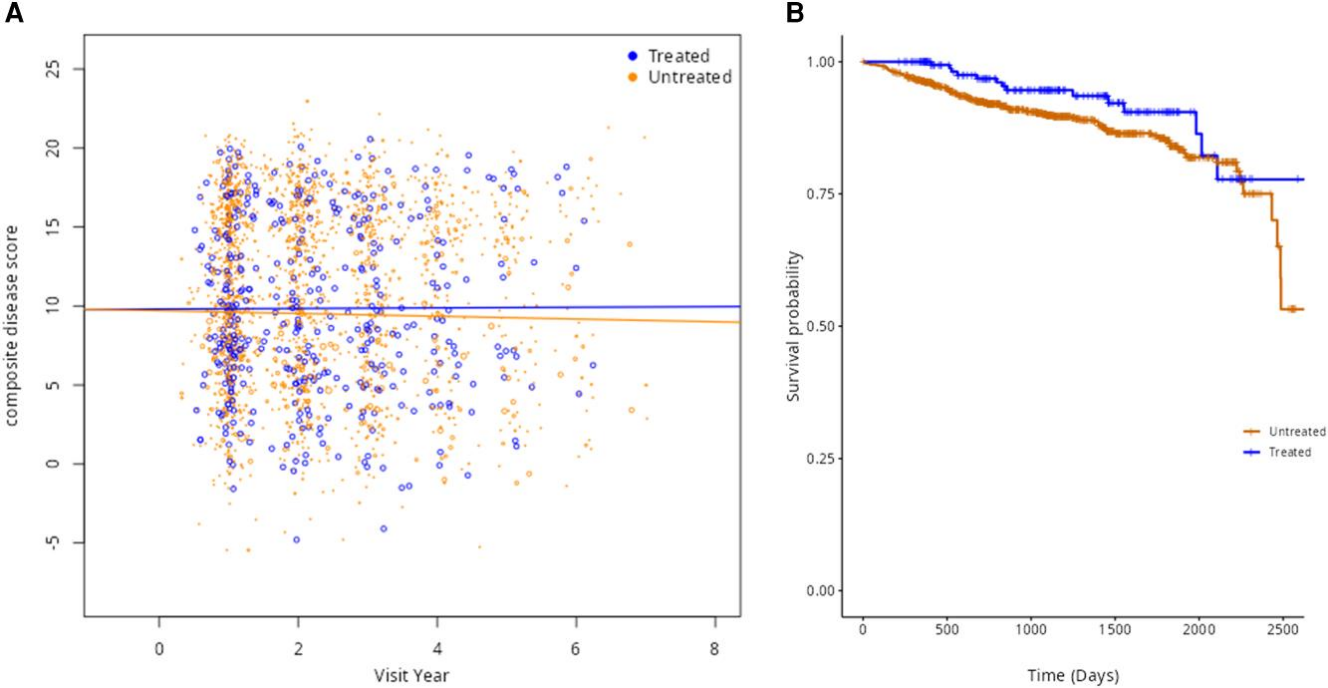
**The effects of antidepressants on disease progression and mortality.** Scatter plot and Kaplan–Meier plot showing the effects on disease progression (**A**) and survival (**B**) of untreated participants (1683/1877) compared with antidepressant treatment (194/1877) following propensity score weighting. Larger circles in **A** denote larger propensity score weights.

Participants experiencing psychiatric symptoms who were treated with antidepressants had slower decline in the composite disease score compared with the untreated group (Fig. 3A and Supplementary Table 10); per year, the composite disease score declined by 0.89 in this group, and antidepressants lessened this decline by 0.36 (95% CI 0.13, 0.6; *P* = 0.002). Likewise, the effect of antidepressant exposure (duration of antidepressant treatment) slowed composite disease score decline: 1 year of treatment with an antidepressant slowed the annual composite disease score change by 0.06 (6.7%/year; 95% CI 0.059, 0.061; *P* = 0.044; Fig. 2B and Supplementary Table 11).

### Examining the effects of antidepressant use on mortality

Mortality rates were similar between the treated and untreated groups (7.2%), but time to death was much longer in the treated group compared with the untreated (1180.93 versus 700.79 days; Supplementary Table 12). Fitting a Cox proportional hazards model showed that this difference was significant: antidepressant use was associated with reduced all-cause mortality risk (HR = 0.38, *P* = 0.04; Fig. 3B and Supplementary Table 13). This effect was not driven by a reduction in suicide rate (Supplementary Tables 14 and 15). The association between antidepressants and reduction in mortality was also seen with antidepressant exposure (HR = 0.66, *P* = 0.043; Supplementary Table 16).

### Effect of antidepressant class on disease progression and mortality

**Figure 4:**
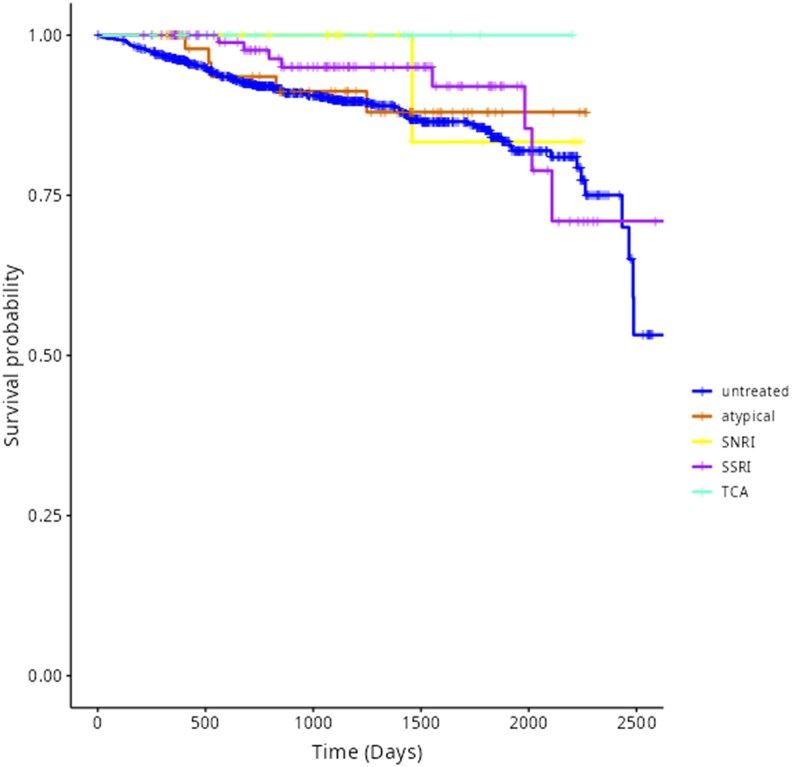
**Survival plot comparing antidepressant classes with no treatment.** Kaplan–Meier plot showing the effect on survival of untreated participants (*n* = 1683) compared with different antidepressant classes following propensity score weighting. SSRI = selective serotonin reuptake inhibitor (*n* = 106); SNRI = serotonin noradrenaline reuptake inhibitor (*n* = 23); TCA = tricyclic antidepressant (*n* = 13); atypical (*n* = 52).

As an exploratory analysis, we looked at the effect of individual antidepressant classes on disease progression in the ENROLL-HD data. We separated antidepressants into tricyclic antidepressants (TCAs), serotonin noradrenaline reuptake inhibitors (SNRIs), selective serotonin reuptake inhibitors (SSRIs) and atypical agents. The majority of study participants received SSRIs (Supplementary Table 17), with relatively small numbers receiving TCAs (*n* = 13). We did not find any individual class effect on composite disease score progression. However, there were significant effects on mortality. Both atypical agents (HR = 0.19, *P* = 0.028) and TCAs (HR = 1.7 × 10^−5^, *P* < 2 × 10^−16^) reduced all-cause mortality (Fig. 4 and Supplementary Table 4) in comparison to no treatment. However, the effects on suicide and non-suicide mortality risk differed: TCAs reduced suicide and non-suicide mortality risk; SSRIs and atypical agents reduced suicide risk alone; and SNRIs affected non-suicide mortality only (Supplementary Tables 18–20). Given that antidepressant class prescribing patterns vary by region,^18^ we created a model also including geographical region (Supplementary Table 21), but this did not change the effect of antidepressant classes on mortality.

### Sensitivity analysis

A tipping-point analysis (Supplementary Fig. 1) of outcome and independent variable showed that, for an unmeasured confounder to eliminate the association between composite disease score and antidepressants, the strength of the association between exposure and outcome would need to be larger than any variable in the model and, in particular, would need to be twice the size of baseline composite score. A sensitivity analysis for an association between an unknown confounder added to the model and the outcome showed that such a confounder added to the model that accounted for >4.98% of the residual variation in composite disease score would eliminate the effect of antidepressant treatment on rate of change of the composite disease score. This is less than the effect of baseline composite disease score and time, but more than comorbidities, psychiatric scores, NCAG, age, sex, history of addiction, antidepressant burden and previous mental health events (Supplementary Table 22).

Sequential hotdeck imputation of missing data did not change the association between antidepressant treatment and composite score (Supplementary Table 23), nor did hotdeck imputation of all variables in the model change the propensity scoring outcome for the mortality analysis.

## Discussion

In this work, we have shown that in HD participants with new episodes of depression or anxiety, antidepressant initiation reduces mortality risk and slows disease progression. An exploratory analysis suggests that the relationship between antidepressants and mortality is not explained by a reduction in suicide risk. Given that psychiatric symptoms are common in neurodegenerative diseases more widely, the effect of antidepressants on disease progression in these disorders merits further study. Significantly, we have also shown that in HD, psychiatric symptoms are associated with faster disease progression and increased mortality.

Previous work in the ENROLL-HD dataset had suggested more rapid disease progression in patients treated with antidepressants.^7^ However, the model in this study did not include relevant mental health variables and did not select participants experiencing incident mental health problems, introducing confounding by indication. More recently, a propensity score approach was used in motor-symptomatic participants in ENROLL-HD.^19^ This study included depression and anxiety scores, NCAG and composite disease score components in the model, with a primary outcome of depression score at first follow-up visit after initiation. They found 86 new users of antidepressants but did not find differences at first follow-up on depression or any measure of disease progression. We looked at disease progression over time, included a substantially larger number of participants and found an association both with disease progression and with a reduction in all-cause mortality risk. A number of studies have also noted an association between antidepressant use and the onset of cognitive decline or diagnosis of dementia,^20,21^ although these studies were not able to account for baseline psychiatric symptom severity. More recent observational work in Alzheimer’s disease has shown an association between TCAs (but not other antidepressants) and worsening cognitive decline, but no association with imaging biomarkers of disease progression.^22^ In Parkinson’s disease, antidepressants were initially linked with a higher risk of disease onset in an observational study,^23^ although animal work in Parkinson’s disease models has shown beneficial effects of antidepressant treatment on alpha synuclein deposition,^24^ and a trial of antidepressants for depression in Parkinson’s disease has included disease progression as a secondary outcome.^25^

To date, no clinical trial of antidepressants in HD (for any indication) has included >30 participants in each arm, and the follow-up has been limited to several months. Randomized controlled trials (summarized by Zadegan *et al*.^26^) of fluoxetine (primary outcome: function, measured by total functional capacity), citalopram (primary outcome: cognition), bupropion (primary outcome: apathy) and a novel agent, PNU-96391A (primary outcome: safety and tolerability) all had follow-up periods <6 months and ≤20 participants in each arm. None showed any benefit on established clinical measures of disease progression, but previous work in HD has shown the need for a substantially larger sample size and longer follow-up.^11^

The association between psychiatric symptoms and disease progression in HD suggests that there might be an additional mechanism leading to disease progression other than that driven by increasing NCAG, represented phenotypically by psychiatric symptomatology. Given that psychiatric symptoms are more frequent in many neurodegenerative diseases compared with the wider population, this raises the intriguing possibility that there is a common mechanism underlying these symptoms across neurodegenerative diseases, potentially opening new therapeutic avenues. Determining what this process might be is difficult, because the mechanism(s) leading to depression are uncertain. Previous work by our group has shown overlap with genetic risk for psychiatric disorders in neurodegenerative disease and the general population.^27,28^ Impairments in neurogenesis have been found in patients with depression in the general population, are known to be rescued by antidepressant treatment, and have been found in R6/1 animal models of HD that can be rescued by fluoxetine treatment.^29^ Hypothalamo-pituitary axis dysfunction has been found in depression in the general population and HD.^30^ Evidence of CNS inflammation has been found in depression,^31^ and also in HD, with some data to suggest modification in imaging biomarkers of disease progression with immunomodulatory treatment.^32^

A strength of our approach has been to address confounding by indication. Study participants with more severe symptoms, necessitating symptomatic treatment, might have worse underlying disease and, consequently, a higher risk of death. Hence, the initiation of symptomatic treatment (e.g. starting an antidepressant) might be a marker of more rapid disease progression rather than a cause of more rapid disease progression. We were able to determine symptoms associated with antidepressant initiation, then compare subsequent disease progression and mortality. Confounding by indication is a significant limitation in previous analyses of psychotropic drug use in dementia and other neurodegenerative diseases, hence conflicting results have been seen from large population analyses. For example, Mo *et al*.^33^ and others^34^ have found higher mortality in patients with dementia receiving antidepressants, but others have shown a reduced risk.^35,36^ Likewise, in Parkinson’s disease evidence of both increased and reduced risk of mortality with antidepressant use has been found.^37,38^

We found differential effects of antidepressant classes on mortality: TCAs were associated with reductions in both suicide and non-suicide related mortality, atypical agents and SSRIs with reduced suicide risk, whereas SNRIs were associated with reductions in non-suicide-related mortality. Previous work by our group^39^ has suggested that atypical agents and SSRIs have a greater effect on depression in HD than do other antidepressant classes, which might reflect their effect on suicide rates. The effect of SNRIs and TCAs on non-suicide mortality are more difficult to explain, although data from Parkinson’s disease suggest that nortriptyline, in particular, might delay disease progression.^40^

There are a number of limitations in this study. Propensity score matching is not able to account for unobserved confounders; however, there is some evidence to suggest that observed and unobserved confounders covary.^41^ Further to this, several recent studies have shown that propensity scoring in observational data replicates the significance and effect sizes reported in randomized controlled trials.^42,43^ Our sensitivity analyses suggested that unobserved or mediating variables would need to be as large or larger than time or baseline composite disease score to influence the results, which seems improbable. We used two scales to determine presence/absence of depression or anxiety, because it is not clear which scale is more reliable in people with HD, and the correlation between the scales, although significant, was only moderate (0.43). The NCAG was slightly shorter in the antidepressant-treated group, despite this group being at a more advanced disease stage on the composite disease score. Previous work suggests that psychiatric symptoms might be even more prevalent in individuals with shorter (and even intermediate) NCAG, which might explain this finding.^44^ The effects of antidepressant classes on all-cause mortality risk should be interpreted with a degree of caution, because the numbers in some groups were small.

## Conclusion

In conclusion, in this work we have shown reduced mortality risk and slower disease progression on clinical, imaging biomarker and fluid biomarkers in HD patients treated with antidepressants compared with untreated patients. This finding might be of wider applicability to other neurodegenerative diseases and raises the possibility of a second mechanism contributing to disease progression in HD.

## Supplementary Material

awag009_Supplementary_Data

## Data Availability

Data from ENROLL-HD are available following completion of a specific data request to CHDI as data controllers (https://www.enroll-hd.org/for-researchers/access-data-biosamples/).
