# Supplementary material for: Exploring the association between antidepressants, progression and mortality in Huntington’s disease
Source: Brain. 2026 Jan 21;149(8):2680–8. doi: 10.1093/brain/awag009 (PMC13431818; doi:10.1093/brain/awag009)
Supplement: awag009_Supplementary_Data [file awag009_supplementary_data.pdf]

SUPPLEMENTARY DATA

Electronic Methods
First, we determined the frequency of antidepressant use in HD: antidepressants were the most frequently prescribed medication to patients with HD in the ENROLL-HD dataset, accounting for over 15% of all drugs prescribed. We categorised all listed indications for antidepressants as 'depression', 'anxiety', 'irritability and aggression', 'apathy', 'sleep', 'psychosis', 'other psychiatric symptom', 'motor symptom of HD', 'pain', 'systemic symptom' or 'uncertain' as outlined below.

Depression
"Adjustment disorder","Adjustment disorder with depressed mood","Adjustment disorder with mixed anxiety and depressed mood", "Affect lability","Affective disorder","Agitated depression", "Antidepressant therapy","Depressed mood","Depression", "Depressive symptom", "Dysthymic disorder", "Grief reaction", "Major depression", "Mood altered","Perinatal depression", "Mood disorder due to a general medical condition","Negative thoughts", "Postpartum depression","Seasonal affective disorder", "Suicidal ideation","Suicide attempt","Suicidal behaviour", "Tearfulness", "Negativism", "Persistent depressive disorder".

Anxiety
"Acute stress disorder","Agoraphobia","Anxiety", "Anxiety disorder","Anxiolytic therapy", "Anticipatory anxiety", "Claustrophobia","Phobia of flying","Mixed anxiety and depressive disorder", "Compulsions", "Generalised anxiety disorder", "Nervousness", "Neurosis", "Obsessive thoughts", "Compulsions", "Obsessive-compulsive disorder", "Obsessive-compulsive personality disorder", "Panic attack", "Panic disorder", "Phobia", "Post-traumatic stress disorder", "Social phobia", "Stress", "Tension", "Obsessive rumination","Social anxiety disorder".

Apathy
"Apathy","Lethargy", "Fatigue"

Irritability
"Aggression","Anger","Irritability", "Anger","Conduct disorder", "Irritability postvaccinal","Intermittent explosive disorder" , "Mood swings", "Impatience", "Affect lability"

Psychosis
"Delusion","Delusional disorder, unspecified type","Hallucination", "Hallucination visual","Jealous delusion", "Psychotic disorder", "Schizoaffective disorder", "Delusions, mixed", "Schizoaffective disorder depressive type", "Schizophrenia", "Schizophrenia paranoid type","Schizophrenia, paranoid type", "Acute psychosis", "Paranoia","Somatic Delusion", "Psychotic behaviour", "Hallucination auditory", "Psychotic disorder due to a general medical condition".

Sleep

"Circadian rhythm sleep disorder","Initial insomnia","Insomnia", "Insomnia related to another medical condition", "Middle Insomnia", "Narcolepsy", "Poor quality sleep", "Sedation", "Sedative therapy", "Sleep disorder", "Parasomnia","Sleep disorder due to general medical condition, insomnia type", "Sleep phase rhythm disturbance", "Sleep disorder", "Sleep disorder due to a general medical condition, insomnia type", "Sleep terror","Somnolence","Rapid eye movement sleep abnormal","Somnolence","Rapid eye movements sleep abnormal".

Other Psychiatric

"Abnormal behaviour","Agitation","Akathisia","Amnesia","Abstains from alcohol", "Alcohol abuse", "Alcohol withdrawal syndrome", "Alcoholism","Attention deficit/hyperactivity disorder", "Attention deficit hyperactivity disorder","Binge eating","Bipolar disorder", "Bipolar I disorder","Bipolar II disorder", "Amnesia","Autism spectrum disorder", "Behaviour disorder","Borderline personality disorder", "Bradyphrenia","Burnout syndrome", "Cyclothymic disorder","Confusional state", "Cognitive disorder", "Conversion disorder","Dementia", "Drug dependence","Depressed level of consciousness", "Delirium","Detoxification", "Disinhibition", "Disorientation", "Disturbance in social behaviour","Disturbance in attention","Drooling", "Drug withdrawal syndrome","Eating disorder", "Drug abuse" ,"Emotional distress","Emotional disorder","Euphoric mood", "Eructation", "Fear","Fear of disease", "Feeling of relaxation", "Gambling","Hyperphagia", "Hyperventilation", "Impulse-control disorder", "Impulsive behaviour","Impulse-control disorder", "Kleptomania","Libido disorder", "Loss of libido","Memory impairment", "Mental disorder", "Mental impairment", "Mood swings", "Mild mental retardation","Mania", "Malaise", "Hypersexuality", "Middle insomnia", "Nicotine dependence", "Nightmare", "Paranoid personality disorder", "Perseveration", "Personality change","Personality change due to a general medical condition", "Personality disorder","Polydipsia psychogenic", "Psychomotor hyperactivity","Psychiatric symptom", "Psychosomatic disease", "Relaxation therapy", "Smoking cessation therapy","Supplementation therapy", "Screaming", "Schizotypal personality disorder", "Somatisation disorder", "Somatoform disorder", "Thinking abnormal", "Trichotillomania","Tachyphrenia", "Tobacco abuse", "Tobacco user".

Motor Symptom of HD

"Bradykinesia","Balance disorder","Cerebellar syndrome","Clumsiness","Chorea", "Dystonia","Dyskinesia", "Hypertonia" ,"Hyperkinesia","Movement disorder", "Muscle relaxant therapy", "Extrapyramidal disorder","Hypotonia","Myoclonus", "Oromandibular dystonia","Parkinsonism","Muscle spasticity" ,"Muscle rigidity", "Periodic limb movement disorder", "Restless legs syndrome", "Torticollis" ,"Tremor","Tardive dyskinesia", "Tic".

Pain

"Abdominal pain","Abdominal pain upper","Analgesic therapy","Abdominal tenderness","Allodynia","Anaesthesia","Arthralgia","Arthritis", "Back pain","Chest pain", "Burning sensation","Complex regional pain syndrome","Facial Neuralgia","Facial pain", "Fibromyalgia", "Headache", "Hernia pain","Epicondylitis", "Gastrointestinal pain", "Arthropathy", "Meralgia paraesthetica", "Migraine", "Migraine prophylaxis", "Musculoskeletal pain", "Neck pain","Myalgia", "Neuralgia", "Occipital neuralgia", "Pain", "Pain in extremity", "Pain management", "Procedural pain","Oral discomfort", "Post herpetic neuralgia", "Psychogenic pain disorder", "Radicular pain","Rib fracture","Sciatica",

”Tension headache”, ”Trigeminal neuralgia”, ”Vulvovaginal pain”.

Systemic Illness
”Abnormal loss of weight”,”Ankylosing spondylitis”, ”Arthritis”,”Antiallergic therapy”,”Autoimmune thyroiditis”,”Asthma”, ”Back injury”,”Bladder disorder”,”Bladder irritation”, ”Bladder spasm”,”Brain neoplasm”, ”Bruxism”,”Brachial plexus injury”,”Carpal tunnel syndrome”, ”Abdominal distension”,”Chills”, ”Chronic fatigue syndrome”,”Cough”, ”Corneal dystrophy”,”Craniocerebral injury”, ”Crohn’s disease”,”Cystitis interstitial”, ”Convulsion”,”Convulsion prophylaxis”,”Cystitis”,”Cyclic vomiting syndrome”,”Dysuria”, ”Dyslipidaemia”, ”Decreased appetite”, ”Dermatitis”,”Dermatitis atopic”,”Diabetic neuropathy”,”Dizziness”, ”Duodenal ulcer”, ”Dyspepsia”, ”Dysaesthesia”, ”Dysphagia”,”Dyspnoea”, ”Enuresis”, ”Essential hypertension”,”Ear infection”, ”Eczema”,”Endometriosis”,”Epilepsy”, ”Fatigue”, ”Fall”, ”Gastrooesophageal reflux disease”,”Gastrointestinal reflux disease”, ”Gastrointestinal disorder”, ”Glaucoma”,”Haemorrhoids”, ”Gastritis prophylaxis”,”Head injury”,”Herpes zoster”, ”Hot flush”,”HIV peripheral neuropathy”, ”HIV infection”, ”Hypertension”, ”Hypermobility syndrome”, ”Hyperhidrosis”,”Hypertonic bladder”,”Irritable bowel syndrome”,”Increased appetite”,”Incontinence”,”Intracranial pressure increased”,”Impaired gastric emptying”, ”In vitro fertilisation”,”Intervertebral disc disorder”,”Intervertebral disc degeneration”,”Intervertebral disc protrusion”,”Hiccups”,”Hiatus Hernia”, ”Joint injury”,”Limb operation”,”Lung neoplasm malignant”,”Macular oedema”,”Meniere’s disease”, ”Menopausal symptoms”, ”Menopause”, ”Menstrual disorder”,”Multiple sclerosis”, ”Mitral valve prolapse”,”Motion sickness”,”Muscle contracture”,”Muscle relaxant therapy”,”Microvascular coronary artery disease” ,”Peripheral swelling”, ”Mononeuropathy”,”Muscle spasms”,”Musculoskeletal stiffness”,”Nausea”,”Nerve compression”,”Nerve injury”,”Neuritis”,”Neuropathy peripheral”, ”Oedema peripheral”, ”Obesity”, ”Osteoarthritis”,”Overweight”, ”Otitis media chronic”, ”Paraesthesia”, ”Periarthritis”,”Partial seizures”, ”Pruritis”,”Psoriasis”, ”Psoriatic arthropathy”, ”Paraesthesiae oral”, ”Prophylaxis against gastrointestinal ulcer”, ”Polyneuropathy”, ”Petit mal epilepsy”, ”Post-traumatic epilepsy”, ”Post viral fatigue syndrome”, ”Premenstrual syndrome”,”Rhinitis allergic”,”Regurgitation”, ”Salivary hypersecretion”, ”Pollakiuria”,”Photopsia”,”Radiculopathy”,”Scoliosis”, ”Simple partial seizures”, ”Syncope”, ”Seasonal allergy”, ”Sensory disturbance”, ”Sinusitis”, ”Sleep apnoea syndrome”, ”Seizure anoxic”, ”Sexual dysfunction”, ”Sjogrens syndrome”,”Spinal osteoarthritis”,”Spinal column injury”,”Spinal column stenosis”, ”Somnambulism”, ”Stress urinary incontinence”,”Upper airway obstruction”,”Upper respiratory tract infection”, ”Surgery”,”Urinary incontinence”, ”Urge incontinence” , ”Urinary tract infection”, ”Urticaria”,”Vascular occlusion” ,”Vertigo”,”Vomiting”,”Seizure”, ”Somatic symptom disorder”, ”Vomiting in pregnancy”,”Temporal lobe epilepsy”, ”Temporomandibular joint syndrome”,”Tonic clonic movements”,”Tinnitus”, ”Underweight”,”Weight control”.

Unclear
”NA”,”Huntington’s disease”,”Hypersensitivity”, ”MISSING”,”NOTAPPL”, ”Off label use”, ”Product used for unknown indication”, ”Prophylaxis”, ”Restlessness”, ”Palliative care”, ”Prophylaxis”,”Nervous system disorder”,”Neurological symptom”,”Motor dysfunction”.

Missing Data
The final dataset included missing rates between 16% and 26% for clinical outcome variables after elimination of implausible outliers (i.e. results lying outside the range of the task or scale):

Composite - 26.9%
Stroop word reading test - 22.2%
Symbol digit modality test - 25.5%
UHDRS motor score - 17.2%
UHDRS total functional capacity - 16.3%

Missingness was lower in the antidepressant treated group (24.29% vs 27.34%). Using a multinomial model from the nnet R package, we found that missingness was not significantly associated with treatment group (AIC 6264, antidepressant treatment: estimate -0.16, p=0.076). We subsequently constructed a linear mixed model, with random intercept and slope, to determine the effect of missingess on composite score progression (Tshown below). This showed that participants with missing values had more severe progression over time. As missing rates were higher in the control group, this suggests that, if anything, the effect of antidepressant treatment on clinical progression is likely to be underestimated. Similarly, in the TRACK-HD data, there was no association between missingness and treatment group, whilst the only association between disease severity biomarkers and missingness was with smaller baseline caudate volume.

Effect of Missingness on Composite Score

	Estimate	Std. Error	t value	p value
(Intercept)	11	0.17	1.7×10^3	$<2\text{x}10^{-16}$
missing data	-1.6	0.28	1.7×10^3	1.2×10^{-8}
visit year	-0.6	0.032	6.9×10^2	$<2\text{x}10^{-16}$
missing data: visit year	-0.26	0.05	6.9×10^2	2.6×10^{-7}

sTable 1: Psychiatric Symptoms Effect on Composite Score

	Estimate	Std. Error	t value	p value
(Intercept)	0.96	0.19	5	5.8×10^{-7}
psychiatric symptoms	-0.026	0.021	-1.2	0.23
visit year	-0.46	0.013	-35	$<2.2 \times 10^{-16}$
psychiatric symptoms: visit.year	-0.062	0.0093	-6.6	3.1×10^{-11}
age	-0.0046	0.00089	-5.2	1.9×10^{-7}
sex (male)	-0.0068	0.016	-0.42	0.67
baseline composite disease score	1	0.0026	3.8×10^{2}	$<2 \times 10^{-16}$
baseline PBA depression	-0.0071	0.007	-1	0.31
baseline PBA irritability	0.0083	0.0042	2	0.046
baseline PBA anxiety	0.0087	0.0067	1.3	0.19
baseline PBA suicide	-0.0048	0.029	-0.17	0.87
number of antidepressants	0.0036	0.0092	0.39	0.7
previous mental health event	-0.0015	0.022	-0.07	0.94
addict	-0.012	0.016	-0.72	0.47
psychoactive drug	-0.037	0.021	-1.8	0.074
NCAG	-0.016	0.0033	-5	7×10^{-7}
comorbidities	0.054	0.02	2.7	0.0067

Pseudo R^2 Marginal: 0.83 Conditional: 0.98

sTable 2: PBA Depression Score Effect on Composite Score

	Estimate	Std. Error	t value	p value
(Intercept)	0.97	0.2	4.9	1.2×10^{-6}
PBA depression	-0.019	0.0073	-2.6	0.0083
visit year	-0.49	0.013	-39	$<2 \times 10^{-16}$
PBA depression: visit year	-0.0055	0.0024	-2.3	0.019
age	-0.0047	0.00092	-5.1	3.3×10^{-7}
sex (male)	-0.0012	0.017	-0.069	0.95
baseline composite disease score	1	0.0027	3.7×10^{2}	$<2 \times 10^{-16}$
baseline PBA depression	0.006	0.009	0.67	0.51
baseline PBA irritability	0.01	0.0043	2.3	0.02
baseline PBA anxiety	0.0066	0.0063	1	0.29
baseline PBA suicide	-0.0033	0.03	-0.11	0.91
number of antidepressants	-0.00087	0.0095	-0.092	0.93
previous mental health event	0.0015	0.023	0.066	0.95
addict	-0.011	0.017	-0.63	0.53
psychoactive drug	-0.053	0.021	-2.5	0.014
NCAG	-0.016	0.0034	-4.7	2.3×10^{-6}
comorbidities	0.057	0.021	2.7	0.0062

Pseudo R^2 Marginal: 0.82 Conditional: 0.98

sTable 3: PBA Anxiety Score Effect on Composite Score

	Estimate	Std. Error	t value	p value
(Intercept)	0.95	0.2	4.8	1.7×10^{-6}
PBA anxiety	-0.016	0.0071	-2.3	0.023
visit year	-0.49	0.013	-39	$<2 \times 10^{-16}$
PBA anxiety: visit year	-0.0039	0.0024	-1.6	0.1
age	-0.0046	0.00092	-5	4.5×10^{-7}
sex .male.	-0.0024	0.017	-0.15	0.88
baseline composite disease score	1	0.0027	3.7×10^{2}	$<2 \times 10^{-16}$
baseline PBA depression	-0.0086	0.0069	-1.2	0.21
baseline PBA irritability	0.01	0.0043	2.4	0.018
baseline PBA anxiety	0.019	0.0084	2.3	0.022
baseline PBA suicide	-0.00024	0.03	-0.0081	0.99
number of antidepressants	-0.002	0.0095	-0.22	0.83
previous mental health event	-0.0022	0.023	-0.095	0.92
addict	-0.011	0.017	-0.67	0.51
psychoactive drug	-0.051	0.021	-2.4	0.017
NCAG	-0.016	0.0034	-4.7	3.3×10^{-6}
comorbidities	0.058	0.021	2.8	0.005

Pseudo R^2 Marginal: 0.82 Conditional: 0.98

sTable 4: Psychiatric Symptoms Effect on All Cause Mortality

	Estimate	Hazard Ratio	Std. Error	z	p value
psychiatric symptoms	0.41	1.5	0.094	4.4	9.4×10^{-6}
visit year	0.25	1.3	0.029	8.6	$<2 \times 10^{-16}$
age	0.037	1	0.0054	6.9	6.4×10^{-12}
sex (male)	0.4	1.5	0.085	4.7	3×10^{-6}
baseline composite score	-0.2	0.82	0.011	-19	$<2 \times 10^{-16}$
baseline PBA depression	0.01	1	0.032	0.31	0.75
baseline PBA irritability	-0.056	0.95	0.017	-3.3	0.0011
baseline PBA anxiety	0.02	1	0.03	0.68	0.5
baseline PBA suicide	0.15	1.2	0.1	1.5	0.14
number of antidepressants	-0.0075	0.99	0.038	-0.2	0.84
previous mental health event	-0.21	0.81	0.11	-1.9	0.056
addict	0.28	1.3	0.085	3.3	0.001
psychoactive drug	-0.079	0.92	0.1	-0.78	0.44
NCAG	0.084	1.1	0.018	4.8	1.7×10^{-6}
comorbidities	-0.21	0.81	0.093	-2.2	0.026

Concordance 0.84

sTable 5: Psychiatric Symptoms Effect on Suicide

	Estimate	Hazard Ratio	Std. Error	z	p value
psychiatric symptoms	0.33	1.4	0.32	1	0.31
visit year	0.063	1.1	0.1	0.63	0.53
age	0.0028	1	0.016	0.17	0.86
sex (male)	1.1	2.9	0.33	3.3	0.00096
baseline composite score	-0.018	0.98	0.045	-0.4	0.69
baseline PBA depression	-0.14	0.87	0.13	-1.1	0.28
baseline PBA irritabilty	-0.21	0.81	0.099	-2.1	0.034
baseline PBA anxiety	0.17	1.2	0.099	1.7	0.097
baseline PBA suicide	0.4	1.5	0.21	1.9	0.061
number of antidepressants	-0.27	0.76	0.17	-1.5	0.12
previous mental health event	1.2	3.2	0.31	3.8	0.00014
addict	0.47	1.6	0.29	1.6	0.11
psychoactive drug	0.65	1.9	0.34	1.9	0.057
NCAG	-0.017	0.98	0.06	-0.29	0.77
comorbidities	-1.1	0.33	0.41	-2.7	0.0074

Concordance 0.76

sTable 6: Psychiatric Symptoms Effect on Non-Suicide Mortality

	Estimate	Hazard Ratio	Std. Error	z	p value
psychiatric symptoms	0.42	1.5	0.098	4.2	2.3×10^{-5}
visit year	0.27	1.3	0.031	8.8	$<2 \times 10^{-16}$
age	0.043	1	0.0057	7.6	2.5×10^{-14}
sex (male)	0.37	1.4	0.089	4.1	3.8×10^{-5}
baseline composite score	-0.22	0.8	0.011	-19	$<2 \times 10^{-16}$
baseline PBA depression	0.027	1	0.033	0.82	0.41
baseline PBA irritability	-0.051	0.95	0.018	-2.9	0.0035
baseline PBA anxiety	0.011	1	0.032	0.35	0.73
baseline PBA suicide	0.08	1.1	0.11	0.7	0.48
number of antidepressants	0.013	1	0.039	0.32	0.75
previous mental health event	-0.36	0.7	0.12	-3.1	0.0023
addict	0.27	1.3	0.089	3	0.0026
psychoactive drug	-0.13	0.88	0.11	-1.2	0.24
NCAG	0.1	1.1	0.018	5.6	1.6×10^{-8}
comorbidities	-0.13	0.88	0.096	-1.4	0.17

Concordance 0.87

sTable 7: PBA Depression Score Effect on Mortality

	Estimate	Hazard Ratio	Std. Error	z	p value
PBA depression	0.074	1.1	0.019	3.9	9.5×10^{-5}
visit year	0.28	1.3	0.026	11	$<2 \times 10^{-16}$
age	0.037	1	0.005	7.4	1.2×10^{-13}
sex (male)	0.42	1.5	0.079	5.3	9.3×10^{-8}
baseline composite score	-0.2	0.82	0.0099	-20	$<2 \times 10^{-16}$
baseline PBA depression	-0.015	0.98	0.031	-0.49	0.62
baseline PBA irritabilty	-0.051	0.95	0.016	-3.2	0.0014
baseline PBA anxiety	0.049	1.1	0.028	1.7	0.08
baseline PBA suicide	0.12	1.1	0.11	1.1	0.26
number antidepressants	0.014	1	0.036	0.39	0.7
previous mental health event	-0.28	0.76	0.1	-2.7	0.0073
addict	0.24	1.3	0.079	3	0.0024
psychoactive drug	-0.079	0.92	0.096	-0.82	0.41
NCAG	0.084	1.1	0.017	5.1	3.9×10^{-7}
comorbidities	-0.28	0.76	0.087	-3.2	0.0015

Concordance 0.85

sTable 8: PBA Anxiety Score Effect on Mortality

	Estimate	Hazard Ratio	Std. Error	z	p value
PBA anxiety	0.04	1	0.021	1.9	0.056
visit year	0.28	1.3	0.026	11	$<2 \times 10^{-16}$
age	0.037	1	0.005	7.4	1.7×10^{-13}
sex (male)	0.42	1.5	0.079	5.3	1×10^{-7}
baseline composite score	-0.2	0.82	0.0099	-20	$<2 \times 10^{-16}$
baseline PBA depression	0.009	1	0.03	0.29	0.77
baseline PBA irritability	-0.046	0.95	0.016	-3	0.0032
baseline PBA anxiety	0.041	1	0.03	1.4	0.17
baseline PBA suicide	0.17	1.2	0.1	1.6	0.1
number of antidepressants	0.027	1	0.035	0.75	0.45
previous mental health event	-0.26	0.77	0.1	-2.5	0.012
addict	0.24	1.3	0.079	3	0.0027
psychoactive drug	-0.074	0.93	0.096	-0.77	0.44
NCAG	0.084	1.1	0.017	5.1	3.8×10^{-7}
comorbidities	-0.28	0.75	0.087	-3.2	0.0012

Concordance 0.85

sTable 9: Efficacy of Propensity Scoring

	Unw Treatment (sd)	Unw Control (sd)	Unw KS	Unw p value	PS Treatment (sd)	PS Control (sd)	PS KS	PS p value
age	52.13 (11.77)	49.91 (13.62)	0.10	0.00	52.13 (11.77)	52.07 (11.74)	0.04	0.57
sex (female)	0.57 (0.49)	0.55 (0.50)	0.02	0.25	0.57 (0.49)	0.57 (0.49)	0.00	0.98
baseline composite score	10.77 (5.11)	11.06 (5.70)	0.11	0.00	10.77 (5.11)	10.05 (4.73)	0.09	0.01
baseline PBA depression	4.43 (3.79)	3.55 (3.50)	0.12	0.00	4.43 (3.79)	4.32 (3.81)	0.04	0.55
baseline PBA irritability	3.15 (3.25)	2.83 (3.25)	0.09	0.00	3.15 (3.25)	2.90 (3.24)	0.05	0.20
baseline PBA anxiety	5.89 (3.97)	5.50 (3.64)	0.06	0.03	5.89 (3.97)	5.88 (3.85)	0.04	0.55
baseline PBA suicide	0.86 (2.37)	0.34 (1.35)	0.09	0.00	0.86 (2.37)	0.67 (2.05)	0.03	0.89
number of antidepressants	2.78 (1.81)	0.79 (1.29)	0.58	0.00	2.78 (1.81)	2.55 (1.57)	0.06	0.13
previous mental health event	0.52 (0.50)	0.32 (0.47)	0.20	0.00	0.52 (0.50)	0.47 (0.50)	0.05	0.20
addict	0.57 (0.49)	0.55 (0.50)	0.03	0.73	0.57 (0.49)	0.52 (0.50)	0.05	0.18
psychoactive drug	0.76 (0.43)	0.53 (0.50)	0.23	0.00	0.76 (0.43)	0.74 (0.44)	0.02	0.99
NCAG	43.08 (2.60)	43.37 (3.35)	0.07	0.01	43.08 (2.60)	43.22 (2.57)	0.02	0.98
comorbidities	0.41 (0.49)	0.32 (0.47)	0.09	0.00	0.41 (0.49)	0.38 (0.48)	0.03	0.76

Unw - unweighted, PS - propensity scoring, KS- Kolmogorov-Smirnov Statistic

Only baseline composite disease score showed a significant difference after PS weighting. Z scoring this in each sample (treated vs untreated) showed treated sample: baseline composite disease score= -0.04, untreated sample: baseline composite disease score= 0.01; a difference of 0.05 which is less than the standardised mean difference of 0.1 associated with significant imbalance in propensity weighted samples.
(Austin PC. Stat Med. 2009 Nov 10;28(25):3083-107)

sTable 10: Antidepressant Treatment Effect on Composite Disease Score

	Estimate	Std. Error	t value	p value
(Intercept)	9.85	5.14	1.92	0.055
antidepressant treatment	-0.77	0.34	-2.28	0.023
visit year	-0.89	0.094	-9.49	$<2 \times 10^{-16}$
antidepressant treatment:visit year	0.36	0.12	3.085	0.00204
age	-0.016	0.023	-0.7	0.49
sex (male)	0.019	0.32	0.058	0.95
baseline composite disease score	1.021	0.048	21.12	$<2 \times 10^{-16}$
baseline PBA depression	0.043	0.055	0.78	0.44
baseline PBA irritability	0.00053	0.056	0.009	0.99
baseline PBA anxiety	-0.045	0.055	-0.82	0.41
baseline PBA suicide	0.11	0.14	0.78	0.43
number of antidepressants	-0.079	0.072	-1.084	0.28
previous mental health event	-0.26	0.32	-0.79	0.43
addict	0.18	0.32	0.57	0.57
psychoactive drug	-0.82	0.4	-2.054	0.04
NCAG	-0.19	0.093	-2.065	0.04
comorbidities	0.33	0.35	0.95	0.34

Pseudo R^2 Marginal: 0.90 Conditional: 0.98

sTable 11: Antidepressant Duration Effect on Composite Disease Score

	Estimate	Std. Error	t value	p value
(Intercept)	9.40	4.92	1.91	0.056
1 year antidepressant treatment	0.041	0.085	0.48	0.63
visit year	-0.72	0.073	-9.83	$< 2x10^{-16}$
1 year antidepressant treatment: visit year	0.06	0.03	2.01	0.044
age	-0.014	0.022	-0.66	0.51
sex (male)	-0.005	0.31	-0.016	0.98
baseline composite disease score	1.019	0.047	21.9	$< 2x10^{-16}$
baseline PBA depression	0.038	0.054	0.71	0.48
baseline PBA irritability	0.011	0.053	0.21	0.83
baseline PBA anxiety	-0.039	0.054	-0.73	0.47
baseline PBA suicide	0.088	0.14	0.63	0.53
number of antidepressants	-0.028	0.076	-0.36	0.72
previous mental health event	-0.22	0.31	-0.720	0.47
addict	0.27	0.31	0.87	0.38
psychoactive drug	-0.89	0.39	-2.28	0.022
NCAG	-0.2	0.09	-2.25	0.024
comorbidities	0.33	0.34	0.99	0.32

Pseudo R^2 Marginal: 0.90 Conditional: 0.96

sTable 12: Mortality Frequency and Time to Event

Antidepressant Treatment	Event Rate	Time to Event (Days)
treated	14/194 (7.22%)	1180.93 sd 601.69
untreated	121/1683 (7.2%)	700.79 sd 703.81

sTable 13: Antidepressant Treatment Effect on All Cause Mortality

	Estimate	Hazard Ratio	Std. Error	z	p value
antidepressant treatment	-0.97	0.38	0.22	-2.1	0.04
age	0.045	1	0.016	1.8	0.067
sex (male)	0.47	1.6	0.22	1.2	0.23
baseline composite disease score	-0.23	0.8	0.028	-6.1	1.2×10^{-9}
baseline PBA depression	-0.012	0.99	0.032	-0.2	0.84
baseline PBA irritability	-0.071	0.93	0.037	-0.92	0.36
baseline PBA anxiety	0.023	1	0.03	0.37	0.71
baseline PBA suicide	0.067	1.1	0.05	0.88	0.38
number of antidepressants	-0.11	0.9	0.084	-0.88	0.38
previous mental health event	0.55	1.7	0.23	1.5	0.14
addict	0.99	2.7	0.23	2.6	0.0086
psychoactive drug	-0.83	0.44	0.31	-1.6	0.1
NCAG	0.044	1	0.056	0.59	0.56
comorbidities	0.12	1.1	0.23	0.3	0.77

Concordance 0.88

sTable 14: Antidepressant Treatment Effect on Suicide

	Estimate	Hazard Ratio	Std. Error	z	p value
antidepressant treatment	-1.6	0.2	0.85	-1.5	0.14
age	0.067	1.1	0.05	1.4	0.17
sex (male)	3.4	29	1.2	1.5	0.13
baseline composite disease score	-0.12	0.89	0.13	-1.2	0.23
baseline PBA depression	0.44	1.5	0.18	1.8	0.072
baseline PBA irritability	-0.64	0.53	0.26	-2.5	0.013
baseline PBA anxiety	-0.42	0.66	0.14	-1.6	0.11
baseline PBA suicide	0.39	1.5	0.18	2.2	0.026
number of antidepressants	0.17	1.2	0.14	0.97	0.33
previous mental health event	4.7	1.1×10^{2}	1.4	1.9	0.052
addict	25	8.1×10^{10}	8×10^{3}	7	3.5×10^{-12}
psychoactive drug	1.1	3.1	1.2	0.69	0.49
NCAG	-0.079	0.92	0.14	-0.66	0.51
comorbidities	1.6	4.9	0.81	1.9	0.063

Concordance 0.98

sTable 15: Antidepressant Treatment Effect on Non-Suicide Mortality

	Estimate	Hazard Ratio	Std. Error	z	p value
antidepressant treatment	-0.82	0.44	0.24	0.53	0.12
age	0.042	1	0.017	1.7	0.096
sex (male)	0.3	1.4	0.24	0.73	0.47
baseline composite disease score	-0.24	0.78	0.03	-6.9	6.7×10^{-12}
baseline PBA depression	-0.031	0.97	0.035	-0.47	0.64
baseline PBA irritability	-0.053	0.95	0.038	-0.65	0.51
baseline PBA anxiety	0.056	1.1	0.031	0.87	0.38
baseline PBA suicide	0.024	1	0.06	0.26	0.79
number of antidepressants	-0.18	0.84	0.1	-1.2	0.23
previous mental health event	0.32	1.4	0.25	0.78	0.44
addict	0.72	2.1	0.25	1.8	0.068
psychoactive drug	-0.67	0.51	0.35	-1.2	0.23
NCAG	0.024	1	0.064	0.3	0.76
comorbidities	-0.079	0.92	0.26	-0.18	0.86

Concordance 0.91

sTable 16: Antidepressant Exposure Effect on All Cause Mortality

	Estimate	Hazard Ratio	Std. Error	z	p value
1 year antidepressant treatment	-0.42	0.66	0.094	-2	0.043
age	0.04	1	0.015	1.6	0.099
sex (male)	0.54	1.7	0.22	1.4	0.15
baseline composite disease score	-0.23	0.79	0.028	-6.3	3.4×10^{-10}
baseline PBA depression	-0.0099	0.99	0.032	-0.17	0.86
baseline PBA irritability	-0.094	0.91	0.038	-1.2	0.22
baseline PBA anxiety	0.014	1	0.029	0.22	0.83
baseline PBA suicide	0.083	1.1	0.05	1.1	0.28
number of antidepressants	-0.17	0.84	0.085	-1.4	0.15
previous mental health event	0.65	1.9	0.23	1.8	0.08
addict	0.9	2.5	0.24	2.4	0.016
psychoactive drug	-0.94	0.39	0.31	-1.9	0.06
NCAG	0.037	1	0.055	0.49	0.62
comorbidities	0.21	1.2	0.24	0.5	0.62

Concordance 0.88

sTable 17: Antidepressant Class Frequency

Antidepressant Class	n	percent
none	1683	89.66
atypical	52	2.77
SNRI	23	1.22
SSRI	106	5.64
TCA	13	0.69

sTable 18: Antidepressant Class Effect on All Cause Mortality

	Estimate	Hazard Ratio	Std. Error	z	p value
atypical	-1.6	0.19	0.41	-2.2	0.028
SNRI	0.2	1.2	0.41	0.25	0.8
SSRI	0.43	1.5	0.31	0.88	0.38
TCA	-11	1.7×10^{-5}	2×10^{3}	-15	$<2 \times 10^{-16}$
age	0.096	1.1	0.019	3.2	0.0015
sex (male)	1.4	4.3	0.27	3.5	0.00051
baseline composite disease score	-0.14	0.87	0.03	-4	5.7×10^{-5}
baseline PBA depression	-0.069	0.93	0.037	-1.3	0.19
baseline PBA irritability	-0.12	0.89	0.043	-1.8	0.078
baseline PBA anxiety	0.2	1.2	0.037	3	0.0028
baseline PBA suicide	-0.13	0.87	0.11	-0.99	0.32
number of antidepressants	0.064	1.1	0.087	0.69	0.49
previous mental health event	0.84	2.3	0.25	2.4	0.016
addict	1.1	2.9	0.25	2.7	0.0066
psychoactive drug	-0.34	0.71	0.35	-0.79	0.43
NCAG	0.15	1.2	0.062	2	0.049
comorbidities	-0.65	0.52	0.29	-1.9	0.064

Concordance 0.85

sTable 19: Antidepressant Class Effect on Suicide

	Estimate	Hazard Ratio	Std. Error	z	p value
atypical	-18	1.1×10^{-8}	3.3×10^{3}	-31	$<2 \times 10^{-16}$
SNRI	0.47	1.6	0.82	0.87	0.39
SSRI	-16	9.4×10^{-8}	1.7×10^{3}	-19	$<2 \times 10^{-16}$
TCA	-11	1.1×10^{-5}	4.2×10^{4}	-14	$<2 \times 10^{-16}$
age	0.21	1.2	0.046	6.9	3.9×10^{-12}
sex (male)	1.4	4	1.1	1.7	0.087
baseline composite disease score	0.038	1	0.13	0.35	0.73
baseline PBA depression	0.36	1.4	0.17	3.3	0.0009
baseline PBA irritability	-0.81	0.44	0.4	-3.4	0.00065
baseline PBA anxiety	-0.14	0.87	0.15	-1.3	0.18
baseline PBA suicide	0.043	1	0.2	0.26	0.79
number of antidepressants	0.48	1.6	0.19	5.3	1×10^{-7}
previous mental health event	2.9	18	1.9	1.6	0.1
addict	18	7.4×10^{7}	2.6×10^{3}	39	0
psychoactive drug	0.42	1.5	1.4	0.43	0.67
NCAG	0.4	1.5	0.15	6.4	2×10^{-10}
comorbidities	-0.69	0.5	0.86	-1.2	0.23

Concordance 0.99

sTable 20: Antidepressant Class Effect on Non-Suicide Related Mortality

	Estimate	Hazard Ratio	Std. Error	z	p value
atypical	-0.72	0.49	0.46	-0.91	0.36
SNRI	-19	6.3×10^{-9}	2.9×10^{3}	-27	$<2 \times 10^{-16}$
SSRI	0.59	1.8	0.35	1.3	0.21
TCA	-18	1.6×10^{-8}	4×10^{4}	-20	$<2 \times 10^{-16}$
age	0.04	1	0.021	1.3	0.19
sex (male)	0.74	2.1	0.3	2.6	0.0094
baseline composite disease score	-0.24	0.79	0.036	-8	9.4×10^{-16}
baseline PBA depression	-0.061	0.94	0.043	-1.2	0.22
baseline PBA irritability	-0.027	0.97	0.042	-0.53	0.6
baseline PBA anxiety	0.13	1.1	0.039	2.3	0.02
baseline PBA suicide	0.062	1.1	0.11	0.85	0.4
number of antidepressants	-0.0063	0.99	0.13	-0.05	0.96
previous mental health event	-0.11	0.89	0.32	-0.35	0.72
addict	0.41	1.5	0.27	1.4	0.17
psychoactive drug	-0.21	0.81	0.36	-0.55	0.58
NCAG	-0.005	1	0.078	-0.071	0.94
comorbidities	-0.86	0.43	0.33	-2.2	0.028

Concordance 0.99

sTable 21: Antidepressant Class Effect on Mortality including Regional Effects

	Estimate	Hazard Ratio	Std. Error	z	p value
atypical	-1.6	0.21	0.42	-2.3	0.021
SNRI	0.23	1.3	0.41	0.3	0.76
SSRI	0.52	1.7	0.32	1	0.31
TCA	-11	1.7×10^{-5}	2.2×10^{3}	-15	$< 2 \times 10^{-16}$
age	0.094	1.1	0.019	3.2	0.0012
sex (male)	1.5	4.6	0.27	3.7	0.0002
baseline composite disease score	-0.14	0.87	0.031	-3.7	0.00026
baseline PBA depression	-0.062	0.94	0.039	-1.2	0.22
baseline PBA irritability	-0.12	0.89	0.045	-1.8	0.071
baseline PBA anxiety	0.2	1.2	0.038	2.9	0.004
baseline PBA suicide	-0.15	0.86	0.12	-1	0.3
number of antidepressants	0.061	1.1	0.087	0.67	0.5
previous mental health event	0.97	2.6	0.26	2.8	0.0051
addict	1.1	3.2	0.26	2.9	0.0044
psychoactive drug	-0.42	0.66	0.36	-0.97	0.33
NCAG	0.15	1.2	0.062	2	0.047
comorbidities	-0.71	0.49	0.3	-1.9	0.058
Europe	1.4	4.2	0.85	1.8	0.071
Latin America	3.8	47	1.1	3.4	0.00064
North America	1.6	5.2	0.87	2.1	0.033

sTable 22: Sensitivity Analysis - ENROLL-HD

	estimate	standard error	df	robustness (%)
baseline composite score	1.02	0.05	945.55	45.84
visit year	-0.89	0.09	490.83	28.68
antidepressant treatment: visit year	0.36	0.12	479.02	4.98
antidepressant treatment	-0.77	0.34	892.65	1.06
NCAG	-0.19	0.09	938.51	0.33
psychoactive drug	-0.82	0.40	929.77	0.30
age	-0.02	0.02	945.86	0.00
sex (male)	0.02	0.02	936.32	0.00
baseline pba depression score	0.04	0.05	926.22	0.00
baseline pba irritability score	0.00	0.06	928.97	0.00
baseline pba anxiety score	-0.05	0.05	924.03	0.00
baseline pba suicidality score	0.11	0.14	926.33	0.00
number of antidepressant	-0.08	0.07	887.13	0.00
previous mental health event	-0.26	0.32	935.62	0.00
history of addiction	0.18	0.32	933.84	0.00
comorbidities	0.33	0.35	921.21	0.00

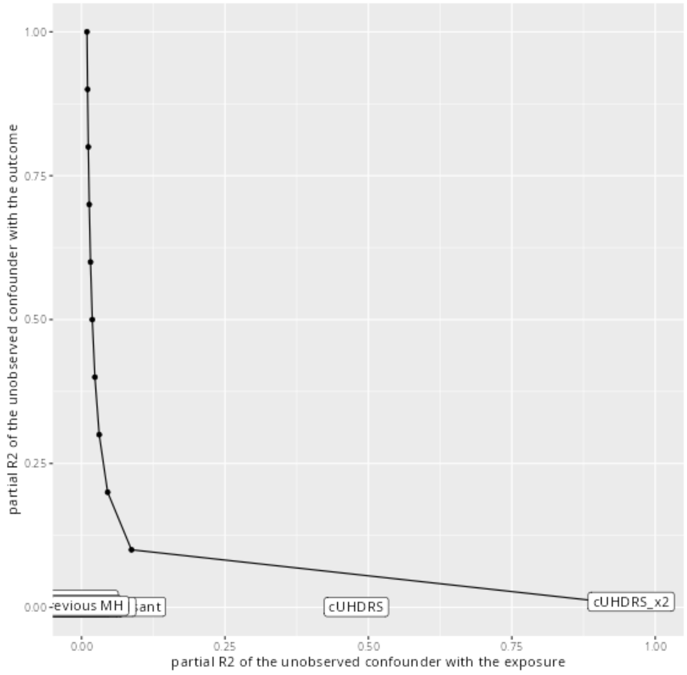

Fig. S1: Tipping Point Analysis Showing the Magnitude of an Unknown Confounder Required to Negate the Effect of Antidepressants on Composite Disease Score in HD.

A confounder twice the magnitude of baseline composite disease score (cUHDRS in the figure) would be required to negate the effect of antidepressants on composite disease score progression.

sTable 23: Antidepressant Effect on Composite Score
Sequential Hotdeck Imputation

	Estimate	Std. Error	t value	p value
(Intercept)	9.84	5.13	1.92	0.06
antidepressant treatment	-0.76	0.33	-2.28	0.022
visit year	-0.89	0.094	-9.49	$< 2x10^{-16}$
antidepressant treatment: visit year	0.35	0.11	3.09	0.002
age	-0.015	0.023	-0.69	0.48
sex (male)	0.018	0.32	0.058	0.95
baseline composite disease score	1.021	0.048	21.11	$< 2x10^{-16}$
baseline PBA depression	0.043	0.054	0.78	0.43
baseline PBA irritability	0.00052	0.056	0.009	0.99
baseline PBA anxiety	-0.045	0.054	-0.82	0.41
baseline PBA suicide	0.11	0.14	0.78	0.43
number of antidepressants	-0.078	0.072	-1.084	0.27
previous mental health event	-0.25	0.32	-0.79	0.42
addict	0.18	0.32	0.57	0.56
psychoactive drug	-0.82	0.4	-2.05	0.039
NCAG	-0.19	0.093	-2.07	0.038
comorbidities	0.33	0.35	0.94	0.34

Pseudo R^2 Marginal: 0.89 Conditional: 0.97
